# Identification of a Splicing Mutation in *ITPR1* via WES in a Chinese Early-Onset Spinocerebellar Ataxia Family

**DOI:** 10.1007/s12311-017-0896-z

**Published:** 2017-12-01

**Authors:** Li Wang, Ying Hao, Peng Yu, Zhenhua Cao, Jin Zhang, Xin Zhang, Yuanyuan Chen, Hao Zhang, Weihong Gu

**Affiliations:** 1 Movement Disorder and Neurogenetics Research Center, Department of Neurology, China-Japan Friendship Hospital, Beijing, 100029 People’s Republic of China; 2 Precisionmdx Inc., 6th Floor, 35 Huayuanbeilu, Beijing, 100083 People’s Republic of China

**Keywords:** Inositol 1,4,5-triphosphate receptor type 1 (*ITPR1*), Nonprogressive cerebellar ataxia (NPCA), Spinocerebellar ataxia type 15/29 (SCA15/29), Whole-exome sequencing (WES)

## Abstract

Mutations in the inositol 1,4,5-triphosphate receptor type 1 gene (*ITPR1*) lead to SCA15, SCA16, and SCA29. To date, only a few families with SCA29 have been reported. A three-generation Chinese family including four affected persons and two unaffected persons were enrolled in this study. We conducted whole-exome sequencing (WES) of the proband DNA initially to find the causal gene. We ascertained the family with autosomal dominant type of congenital nonprogressive cerebellar ataxia (CNPCA) associated with delayed motor and cognitive impairment. WES study was performed with two patients and identified c.1207-2A–T transition, in exon 14 of *ITPR1*, which was a splicing mutation. Sanger sequencing showed that four patients within this family carried the mutation and two unaffected members did not carry it. The results showed that the novel splicing mutation of *ITPR1* was the causative gene for SCA29. In conclusion, we identified a novel SCA29 causative splicing mutation of *ITPR1* in a Chinese family. We suggest *ITPR1* gene analysis shall be a priority for diagnosis of patients with early-onset CNPCA. Our study demonstrated that whole-exome sequencing might rapidly improve the diagnosis of genetic ataxias.

**Electronic supplementary material:**

The online version of this article (10.1007/s12311-017-0896-z) contains supplementary material, which is available to authorized users.

## Introduction

The spinocerebellar ataxias (SCAs) are known as a group of genetically heterogeneous disorders. Mutations in the inositol 1,4,5-triphosphate receptor type 1 gene (*ITPR1*) lead to SCA15 [1], SCA16[2], and SCA29 [3, 4]. SCA15 is an autosomal dominant, late-onset, very slowly progressive pure cerebellar ataxia, and SCA16 has been known to be essentially similar to SCA15. SCA29 is also an autosomal dominant, pure cerebellar ataxia caused by an *ITPR1* mutation, but with early-onset. Thus, SCA15 and SCA29 are genetically identical disorders showing very slowly progressive or pure nonprogressive cerebellar ataxia (NPCA). Mutations in the *ITPR1* gene are associated with slowly progressive cerebellar ataxia. Here, we report a Chinese family mapped to the SCA15/29 locus that has a novel splicing mutation, c.1207-2A>T transition, in exon 14 of *ITPR1*, which was identified by whole-exome sequencing.

## Methods

### Subjects

**Fig. 1:**
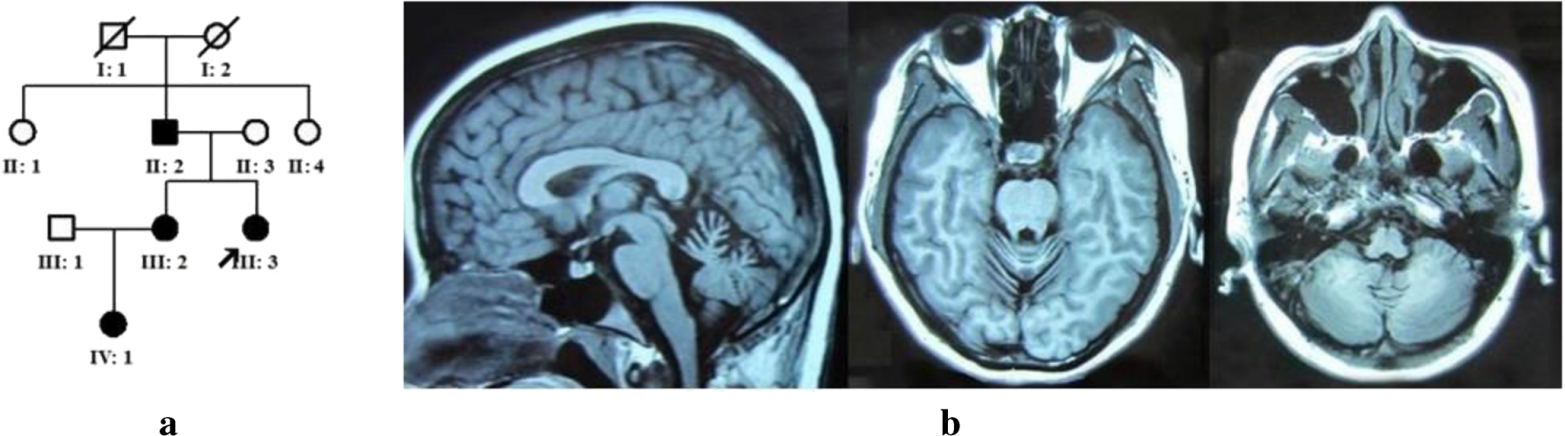
Pedigree charts of the family and brain magnetic resonance imaging (MRI) of III:2. **a** Pedigree charts of the family. Squares indicate males; circles indicate females. Affected individuals are indicated by solid symbols, and unaffected individuals by open symbols. Arrow points to proband. **b** MRI of III:2 indicated cerebellar vermis atrophy

A non-consanguineous Chinese family including four affected persons (proband, father, sister, and niece), mother (unaffected), and two aunts (unaffected) was enrolled in this study (Fig. 1a). Among four patients, dominant inheritance history of congenital nonprogressive cerebellar ataxia (CNPCA) associated with delayed motor milestone and cognitive impairment was observed. Genomic DNA was extracted from peripheral EDTA-treated blood using blood genomic extraction kit (Qiagen, Germany) and quantified using a Nano Drop 2000 unit (Thermo Fisher Scientific, Wilmington, DE). This study was approved by the Ethics Committee of China-Japan Friendship Hospital. The methods in this study were performed in accordance with the approved guidelines. Written informed consent was obtained from all the subjects. All the patients underwent a standard neurologic examination conducted by two qualified neurologists.

### Whole-Exome Sequencing

We conducted whole-exome sequencing (WES) of the proband DNA initially to find the causal gene. Library was prepared using 3 μg of high molecular weight genomic DNA as the starting material using the Agilent SureSelect Library Prep kit. A 300-400-bp library, including adaptor sequences, was successfully obtained. Agilent SureSelect Human All Exon V5 kit was used to perform capture. After adding barcode, this enriched library was sequenced on a Hiseq 2500 platform (Illumina, San Diego, CA) for paired-end reads of 100 bp at Precisionmdx. Co. (Beijing, China; http://www.precisionmdx.com/). The reads were aligned against the human reference genome (hg19) using the SOAPaligner program (http://soap.genomics.org.cn/soapaligner.html). The single-nucleotide polymorphisms (SNPs) were identified using the SOAPsnp program (http://soap.genomics.org.cn/soapsnp.html). Subsequently, the reads were realigned to the reference genome by the BWA program, and insertions or deletions (InDels) were identified with the GATK program (http://www.broadinstitute.org/gsa/wiki/index.php/Home_Page). Then, the identified SNPs and InDels were annotated using the Exome-assistant program (http://122.228.158.106/exomeassistant). Allele frequency and reported pathogenicity were assessed using the dbSNP138, Human Gene Mutation Database (HGMD) [5], 1000 Genomes Project, and Exome Sequencing Project (ESP6500). Variant pathogenicity was assessed using ACMG guidelines [6]. The effect of single-nucleotide variants (SNVs) will be predicted by SIFT, Polyphen-2, and Mutation Taster programs. The candidate clinically significant causal variants identified via WES were further confirmed by means of Sanger sequencing. Co-segregation analyses were also conducted with samples from other family members.

## Results

### Clinical Features

Clinical features are illustrated in the Table 1. There are four affected individuals in the three-generation Chinese family (Fig. 1a). All the affected individuals have history of delayed walking and speaking and a wide-based gait in childhood with an unremarkable perinatal history. In addition, they all have learning difficulties at school and dropped out after primary school education. After 10 years old, they have hard time to keep balance as they often fall even at flat surface. All the patients showed motor developmental delays, cerebellar ataxia, postural and action tremor of the hand, dysarthria, and mild cognitive deficits. Muscle stretch reflexes decreased. Pyramidal features were negative. Muscle strength and autonomic nervous system were normal. Horizontal nystagmus was observed in the proband (III:3). Brain MRI of III:2 and III:3 showed cerebellar vermis atrophy without brainstem and cerebellar hemisphere atrophy (Fig. 1b). The vertical mode of inheritance is consistent with autosomal dominant inheritance.

**Table 1:** Clinical features of affected individuals in the family

Affected individual	II:2	III:2	III:3	IV:1
Age at onset, years	From infancy	From infancy	From infancy	From infancy
Age at speak, years	3	2	4	2
Age at walk, years	6	5	6	5
Age at examination, years	51	29	24	6
Gaze nystagmus	Unknown	−	+	−
Dysarthria	+	+	+	+
Truncal and limb ataxia	+	+	+	+
Reflexes				
Upper limb	↓	↓	↓	↓
Lower limb	↓	↓	↓	↓
Postural tremor	+	+	+	+
Action tremor	+	+	+	+
Gait disturbance	+	+	+	+
Pyramidal features	−	−	−	–
Cerebellar atrophy	Unknown	CV	CV	Unknown
MMSE	Unknown	26	23	Unknown

*CV* cerebellar vermis

+ positive; − negative; ↓ decrease

### Genetic Findings

**Fig. 2:**
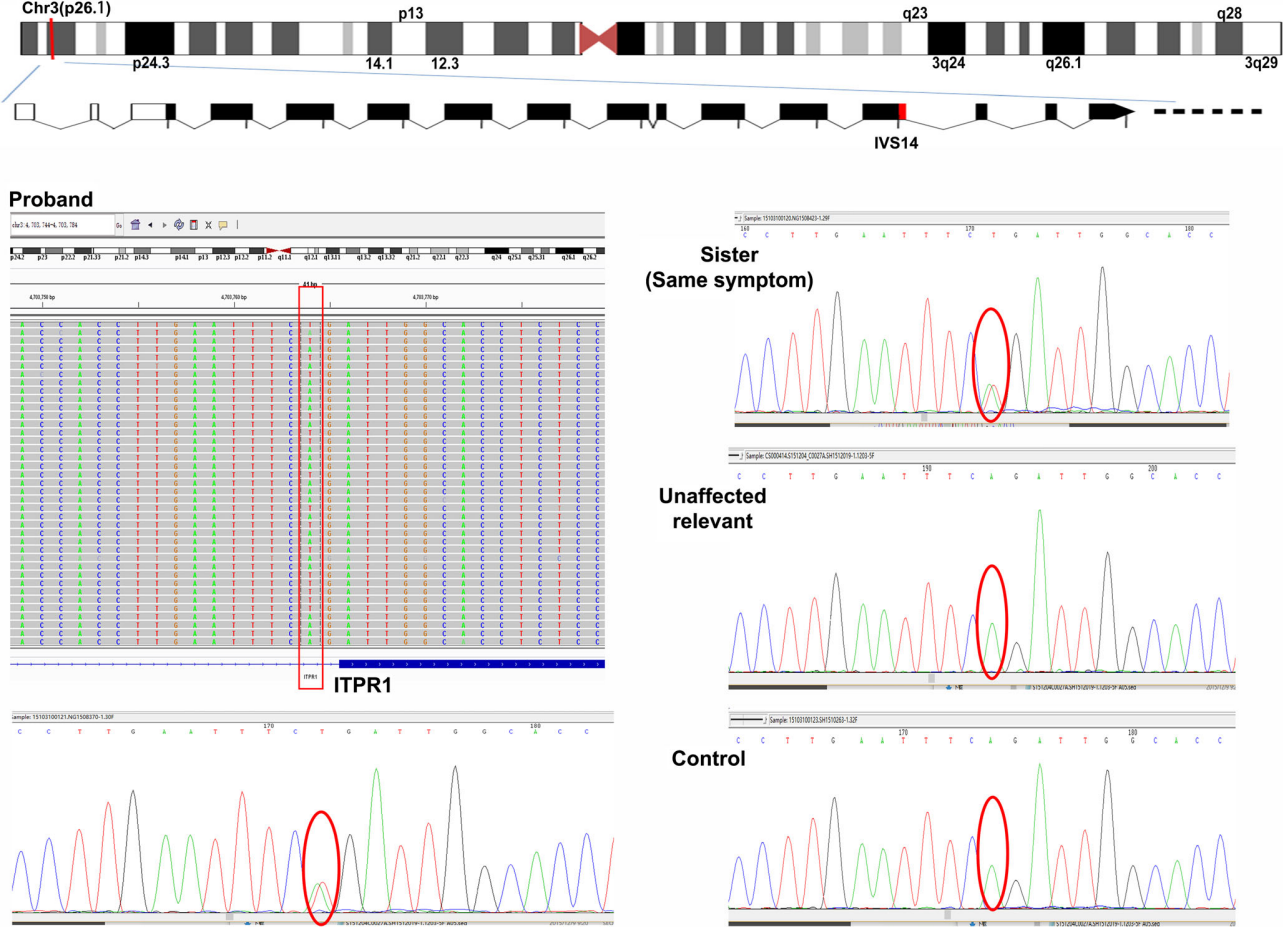
Heterozygous splicing mutations in ITPR1 in this family. Segregation of mutation site (c.1207-2A>T) with Sanger sequencing is represented by oval. Affected individuals are indicated by gray pillar. Sequence traces from control, unaffected, and an affected member of this family show the heterozygous mutation c.1207-2A>T (red) in the affected individuals

Abnormal expansions of trinucleotide repeats in the *SCA1*, *2*, *3*, *6*, *7*, *8*, *12*, and *17* and *DRPLA* gene, or ATTCT repeats of the *SCA10* gene were excluded (data not shown). Whole-exome sequencing was performed. After fastq file was mapped to human genome sequencing, VCF file was generated. Total of 18,293.08-Mb data was achieved, with average sequencing depth of 163.57×; 93.9% of whole exome were covered at least 20×. It indicates good data quality. We analyzed and interpreted WES data through HGMD, 1000 Genomes, inhouse, virtual panel, SIFT, Polyphen2\MutationTaster, and GERP++; 85,768 variants were found. We then went through HGMD database and found 87 variants. Because dominant inheritance is observed in the family, we filtered variants through 1000 Genomes and inhouse databases, 15 variants were found (MAF < 0.0001). However, none of them are associated with core phenotype (ataxia and cerebellar atrophy). Therefore, we have all 85,768 variants went through virtual panel, which was designed by selecting 577 genes from OMIM, HPO, Mingjian, and Neuromuscular Disease Center web information. Among the 577 genes, 516 were reported to be associated with ataxia and 237 with cerebellar atrophy. We found 2216 variants that reside in those 577 genes. We again had these variants went through 1000 Genomes and inhouse databases and found 30 qualified variants (MAF < 0.0001). There are nine of them associated with both ataxia and cerebellar atrophy, including three same sense mutations, two mutations with unknown phenotype, one splicing mutation, one frameshift mutation, one insertion in intron region, and one missense mutation. Following dominant inheritance principal and ACMG guidelines, *ITPR1*c.1207-2A>T splicing and SPTBN2 c.6535 del frameshift are potential causative mutations. We further analyzed detailed clinical symptoms and decided to test mutation on *ITPR1*. We sequenced family members with Sanger sequencing on this mutation and found that results are in accordance with clinical symptoms observed among family members (Fig. 2). This mutation has never been reported. The above process is described in supplementary figure.

## Discussion

In the previously reported families with autosomal dominant nonprogressive cerebellar ataxia (NPCA) [4, 7], mild to moderate early-onset NPCA, delayed motor milestones, and cerebellar vermal hypoplasia on imaging are consistent features. Although cognitive impairment is regarded as a characteristic feature of NPCA [8], only half of those families have cognitive impairment. In this Chinese family, the vertical mode of inheritance is consistent with autosomal dominant inheritance. Four patients of this family have a homogeneous phenotype and were characterized by CNPCA, motor developmental delays, postural and action tremor of the hand, dysarthria, and mild cognitive deficits. This phenotype is very similar to that observed in SCA29 [9]. The family showed co-segregation of the mutation with the phenotype and its absence in normal unaffected individuals.

*ITPR1* is known as causative gene of SCA including SCA15 and SCA29 [9]. SCA15 is a rare, autosomal dominant, adult-onset, relatively slowly progressive ataxia with normal cognition [1]. Cerebellar atrophy, particularly of the vermis, is the typical neuroradiological feature [10]. SCA15 was first reported in 2001 [11], the locus mapping to chromosomal region 3p24.2-3pter [12, 13]. In 2007, partial deletions of the *ITPR1* gene on the distal short arm of chromosome 3 was identified to cause SCA15 development [14, 15]. In 2001, Miyoshi et al. [16] reported a four-generation Japanese family with autosomal dominant spinocerebellar ataxia. Head MRI demonstrated cerebellar atrophy without brainstem involvement. Mutation analysis by PCR excluded mutations in previously identified genes causing SCA. Then, the disorder was designated as SCA16. Miura et al. [17] provided follow-up on this family and found that the contactin 4 gene locus at 3p26 is a candidate gene of SCA16. In 2008, Iwaki et al. [2] identified a heterozygous deletion of exons 1–48 of *ITPR1* in SCA16 which indicated that SCA15 and SCA16 are the same disorder, due to haploinsufficiency of *ITPR1*.

In 2004, Dudding et al. [4] reported a four-generation Australian kindred of Caucasian ancestry with SCA29. All affected individuals had congenital onset of ataxia or delayed walking and wide-based gait as a young child with cognitive impairment of varying degrees. By genome-wide screen of a member of the family detected linkage to chromosome 3p with a maximum two-point lod score of 4.26 at D3S3630. The disease locus lies distal to D3S1304 in the pter region of chromosome 3. Approximately 8 cM of the candidate region overlaps with the locus defined for SCA15. However, their phenotypes are different. SCA29 is distinguished from SCA15 by onset in infancy of delayed motor development followed by NPCA and mild cognitive impairment. Additional variable features include nystagmus, dysarthria, and tremor [3]. In 2012, Huang et al. [3] reported missense mutations in *ITPR1* gene on chromosome 3p26-p25, which cause SCA29.

An *ITPR1* point mutation was discovered in one family with SCA15 [18] and two families with SCA29 [3]. The expression level of *ITPR1* mRNA and protein in SCA15 patients are lower than those in control subjects [14, 18, 19]. The missense mutation associated with SCA15 reduces the level of IP3R1 protein expression [20], resulting in reduced IP3R-mediated Ca^2+^ influx in the central nervous system and particularly in cerebellar Purkinje cells [10], which leads to persistent long-standing dysfunction of Purkinje cells and eventually degeneration of selective neuronal populations. Meanwhile, the molecular mechanisms responsible for SCA29 are poorly understood. We demonstrate in this study that a novel *ITPR1* heterozygous mutations c. 1207-2A>T exists in this SCA29 family, splicing site that leads to rearrangements in *ITPR1*. Further analysis is required to understand functional effects of this mutation in SCA29.

In clinical practice, cerebellar ataxia, mental retardation, dysequilibrium syndrome and pontocerebellar hypoplasia present the similar phenotype with early-onset. However, both of them are passed on from generation to generation in line with autosomal recessive inheritance pattern.

Whole-exome sequencing is a diagnostic test for patients with nonspecific or unusual disease presentations of possible genetic cause [21]. Now, WES is widely used to identify causative genetic mutations of diseases [22]. For more definitive diagnosis, WES has been increasing applied to patients with cerebellar ataxia or cerebellar atrophy [23–25]. WES on an Illumina HiSeq 2500 platform also helped us to reveal a new mutation in causative genes for SCA29. Also, the findings of novel potential pathogenic variations provided inherited clues for further functional research. This study suggests that performing WES on affected individuals is an effective and cost-efficient method for mapping genes of rare Mendelian disorders.

ACMG guidelines [6] are strictly followed when variants were interpreted. This discovered variant, c.1207-2A>T of *ITPR1* gene is a splice site mutation 2-bp upstream of exon 14, which would result in complete skipping of the exon 14 and loss of *ITPR1* function. Several cases of *ITPR1* loss of function mutations have been reported to be pathogenic [2, 26, 27], so c.1207-2A>T of *ITPR1* gene meets condition of the ACMG guidelines as very strong pathogenic evidence (PVS1). In addition, this mutation is absent in 1000 Genomes (www.1000genomes.org), ESP6500 (evs.gs.washington.edu/EVS/), and ExAc (exac.broadinstitute.org/) databases. Therefore, the moderate pathogenic evidence (PM2) is also satisfied. The clinical features of disease caused by mutation of *ITPR1* gene are highly consistent with this case. The c.1207-2A>T mutation is segregated within the family of the proband. This also supports pathogenic evidences (PP1 and PP4). Based on these facts, c.1207-2A>T of ITPR1 meets conditions of four pathogenic evidence (PVS1, PM2, PP1, and PP4), which should be categorized to be pathogenic according to the ACMG guideline of sequence varaints [6].

In conclusion, we identified a novel SCA29 causative splicing mutation of *ITPR1* in a Chinese family. We suggest *ITPR1* gene analysis shall be a priority for diagnosis of patients with early-onset CNPCA. WES is an efficient tool to analyze potential mutations.

## Electronic supplementary material

ESM 1 (DOCX 149 kb)
