# Supplementary material for: Identification of a Splicing Mutation in ITPR1 via WES in a Chinese Early-Onset Spinocerebellar Ataxia Family
Source: Cerebellum. 2017 Dec 1;17(3):294–9. doi: 10.1007/s12311-017-0896-z (PMC5966481; doi:10.1007/s12311-017-0896-z)
Supplement: Supplementary file 1 — (DOCX 149 kb) [file 12311_2017_896_MOESM1_ESM.docx]

**Supplememental Figure: Targeted gene mutation screen complete analysis pipeline**
